# Perceptual judgments of duration of parabolic motions

**DOI:** 10.1038/s41598-021-86428-3

**Published:** 2021-03-29

**Authors:** Björn Jörges, Barbara La Scaleia, Joan López-Moliner, Francesco Lacquaniti, Myrka Zago

**Affiliations:** 1 Center for Vision Research, York University, Toronto, ON Canada; 2 Laboratory of Neuromotor Physiology, IRCCS Fondazione Santa Lucia, Via Ardeatina 306, 00179 Rome, Italy; 3 Vision and Control of Action (VISCA) Group, Department of Cognition, Development and Psychology of Education, Institut de Neurociències, Universitat de Barcelona, Barcelona, Catalonia Spain; 4 Department of Systems Medicine and Centre of Space Bio-Medicine, University of Rome Tor Vergata, 00133 Rome, Italy; 5 Department of Civil Engineering and Computer Science and Centre of Space Bio-Medicine, University of Rome Tor Vergata, 00133 Rome, Italy

**Keywords:** Neuroscience, Perception

## Abstract

In a 2-alternative forced-choice protocol, observers judged the duration of ball motions shown on an immersive virtual-reality display as approaching in the sagittal plane along parabolic trajectories compatible with Earth gravity effects. In different trials, the ball shifted along the parabolas with one of three different laws of motion: constant tangential velocity, constant vertical velocity, or gravitational acceleration. Only the latter motion was fully consistent with Newton’s laws in the Earth gravitational field, whereas the motions with constant velocity profiles obeyed the spatio-temporal constraint of parabolic paths dictated by gravity but violated the kinematic constraints. We found that the discrimination of duration was accurate and precise for all types of motions, but the discrimination for the trajectories at constant tangential velocity was slightly but significantly more precise than that for the trajectories at gravitational acceleration or constant vertical velocity. The results are compatible with a heuristic internal representation of gravity effects that can be engaged when viewing projectiles shifting along parabolic paths compatible with Earth gravity, irrespective of the specific kinematics. Opportunistic use of a moving frame attached to the target may favour visual tracking of targets with constant tangential velocity, accounting for the slightly superior duration discrimination.

## Introduction

According to a venerable concept, the brain has evolved to exploit physical invariants of the environment by internalizing some of their relevant features^1^. Internal representations may or may not mirror the corresponding physical properties, but they often preserve the information most essential for the specific task at hand while ignoring irrelevant information^2^. The specific nature of the internal representation for a given environmental property may vary greatly as a function of the task and context. Accordingly, it has been proposed that there exist different knowledge systems for reasoning and acting on mechanical systems governed by Newton’s laws^3–6^.

This appears to be the case for the representations of projectile motion, that is, the motion of objects (such as a ball) initially thrown forward and upward with an impulsive force, then proceeding along a parabolic path under the constant effect of gravity (plus, possibly, the effect of air resistance). Predictions for acting on projectiles are linked with real physics, but explanations or judgments about their motion are susceptible to biases^5^. It has been argued that manual interception and ocular pursuit of projected balls are based on internalized gravity effects^7,^^8–11^ plus on-line visual cues^12–14^. The role of internalized gravity has been established in an especially compelling way when large segments of the parabolic trajectory were occluded from view, yet people timed interceptions with the same accuracy as under full view^15,16^. On the contrary, the cognitive representations of parabolic trajectories often reflect naïve rather than Newtonian physical conceptions^4,5,17^. Thus, when people provide verbal or pictorial responses, they often misjudge the shape and kinematics of the trajectories, as well as the landing position of the target^18–22^. Despite the erroneous beliefs, people tend to be confident about their responses, showing that the cognitive representation of ball motion is subjectively reliable.

Here, we address the issue of the internal representation for the discrimination of motion duration. Previous work showed that a gravity constraint is taken into account in the discrimination of motion duration for targets shifting along the vertical^23–25^. These studies found that the discrimination precision was higher when the target accelerated downwards than when it accelerated upwards, consistent with a constraint that specifies downward motion for targets accelerating under gravity. However, the results held irrespective of the specific value of gravity (Earth gravity^24,^^23^, Earth or Mars gravity^25^) or the semantic nature of the accelerating object (ball versus rocket^23^. Since these studies only employed accelerating profiles along a linear path in the fronto-parallel plane, they could not discriminate between a representation based on internalized Newton’s laws (such as that used in target interception) and a heuristic representation based on a spatial template of the trajectory (downward motion under gravity). Both kinds of representation would incorporate physical principles, but the former would fully abide by Newton’s laws in the terrestrial gravitational field, whereas the latter would only incorporate a subset of the physical constraints.

One way to discriminate between these two possibilities consists in using projectiles approaching the observer along the sagittal plane in a virtual reality scenario. There are several reasons why this kind of stimuli poses specific challenges to the observer as compared with motion stimuli presented in the fronto-parallel plane. In the following, we assume that the observer is stationary and keeps fixation on a given point along the target trajectory, rather than tracking the target with gaze shifts (detailed description of gaze-tracking strategies for projectile balls can be found, for example in^26^). In general, the recovery of spatio-temporal information is more complex for in-depth motion than for motion in the fronto-parallel plane^14^. A projectile shifts along a parabolic path (in the absence of air drag) when viewed in a fronto-parallel plane. By contrast, the projectile appears as shifting along a straight path when viewed in the sagittal plane, and the time when it reaches the highest position depends on the distance between the observer and the landing point. Moreover, both the mapping of target speed from distal (world) to proximal (retina) coordinates and the available visual cues differ between the two projection planes^27,28^. For fronto-parallel motion, speed on the retina is proportional to speed in the world, with the exact proportion depending on the distance to the observer. However, the relationship is non-linear during in-depth motion; even in the case of an object approaching the observer along the sight-line at constant world speed, the resulting image on the retina would be accelerating. As for the visual cues used to determine target speed, the major cue for fronto-parallel motion comes from retinal image translation. For motion in depth, both monocular (optic flow) and binocular (changing disparity and inter-ocular velocity difference) cues can contribute^27^. Even with both sets of cues available, speed discrimination of in-depth motion is significantly worse than the speed discrimination of fronto-parallel motion^28^. Another fundamental difference pertains to the ecological nature of the task. In the case of fronto-parallel trajectories, participants have to judge distant events. In the case of approaching projectile motions, the task places participants in an immersive context, close to daily-life situations such as those of ball catching.

**Figure 1:**
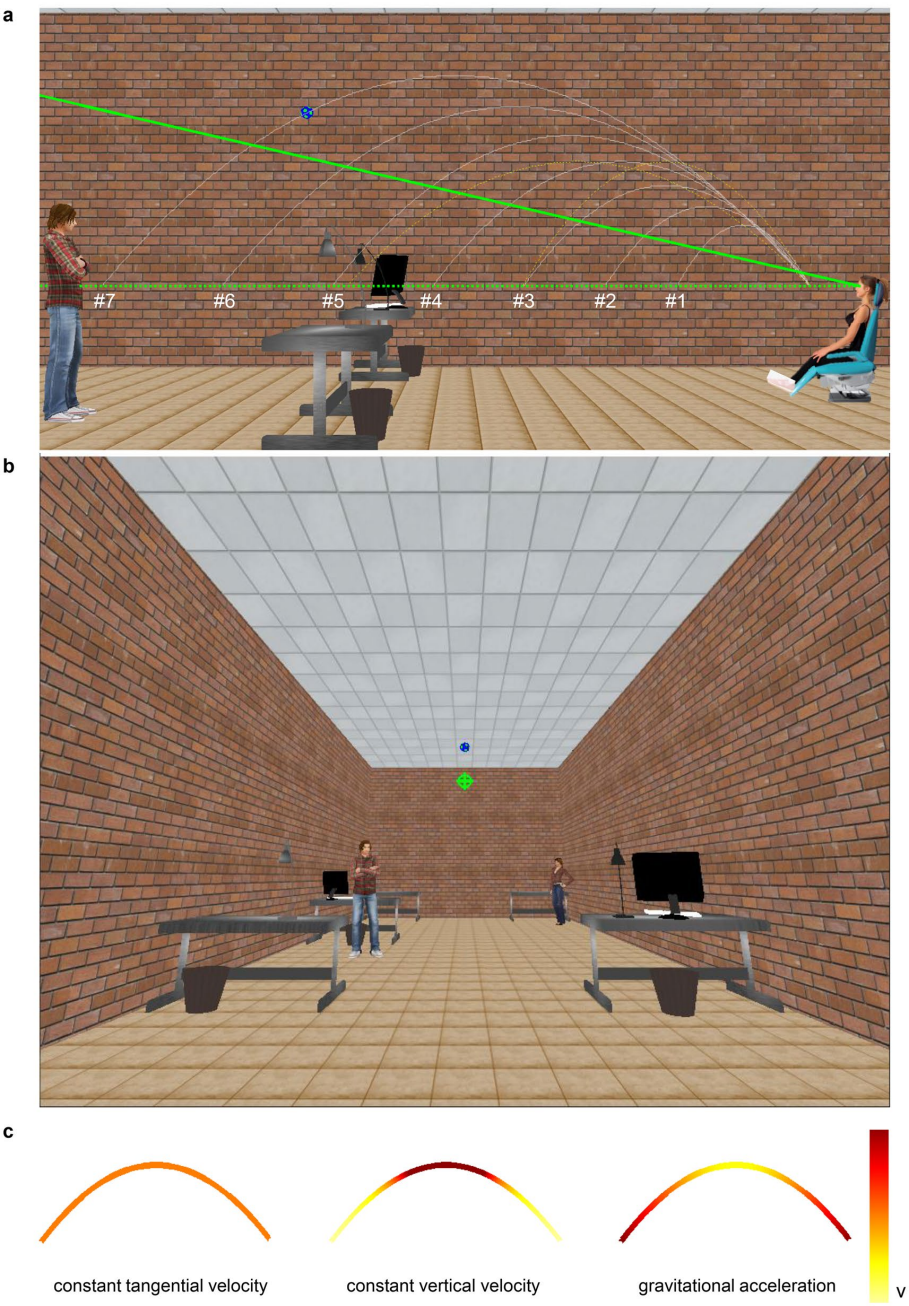
Schematic of the experimental setup and visual stimuli. Panel (**a**) The seated woman and the blue chair indicate the position of the participant. White lines represent the parabolic paths of the target (textured blue ball) for conditions #1-#7. Yellow dot lines represent the parabolic paths for the control conditions #8 and #9. Starting position and final position of the ball are aligned with participant’s eyes (green horizontal dotted line). Solid green line is the gaze line during fixation. Panel (**b**) Visual stimulus as seen by the participant. The fixation cross is green. Panel (**c**) Exemplary profiles of color-coded changes of the tangential velocity for the three laws of motion. Velocity increases from yellow to red in the color scale.

We used visual stimuli obeying the spatio-temporal constraint of parabolic motion under Earth gravity, and either obeying or violating the corresponding kinematic constraint. Target balls approached the observer in the sagittal plane along different trajectories in an immersive 3D virtual scenario (Fig. 1a,b). In each trial, the observer was asked to judge whether the target took longer to arrive at destination (at 0.5 m from the observer) during a reference trajectory with fixed duration or during a comparison trajectory with variable duration, these two stimuli being presented sequentially. Both the reference and the comparison obeyed one of three possible kinematic laws, randomly interleaved across trials: constant tangential velocity, constant vertical velocity, or acceleration due to Earth gravity (Fig. 1c). Only the motion with gravitational acceleration was fully consistent with Newton’s laws in the terrestrial gravitational field, with the target decelerating in the ascending part of the parabolas and accelerating in the descending part. The motions with constant velocity profiles deviated drastically from gravitational motion throughout the trajectory. However, target paths for all stimuli were designed to be compatible with both the curvature profile and the duration of motion under gravity. We asked participants to maintain fixation instead of tracking the target with the eyes to eliminate the potential confound of different eye movement strategies as a function of target kinematics. In fact, it is known that eye-tracking errors are significantly smaller for ballistic trajectories under gravity than for constant speed trajectories^8^ or other non-natural kinematics^10^. Therefore, the presence of gravitational acceleration in the currently tested parabolas might have, in principle, favoured the corresponding perceptual judgments of motion duration just because these trajectories were better tracked by eye movements, if the latter had been permitted.

The hypothesis of a quantitative internal model of gravity effects predicts a better discrimination of duration of motions under gravitational acceleration. By contrast, the hypothesis of a heuristic model based on a spatio-temporal template of the trajectory predicts that the duration discrimination of the trajectories should be comparable irrespective of the specific kinematics, since the path was identical in all conditions. In the extreme case, opportunistic judgements might favour motions with constant velocity profiles, since these profiles might be easier to track visually, so that their duration might be discriminated better than for decelerating/accelerating profiles.

## Methods

### Participants

We recruited 17 participants (9 females and 8 males, age range 24–48 years) for the experiment. Sample size was calculated to detect an effect size of 0.95 (Cohen’s d, estimated from^24^ and pilot data with the current setup), by considering paired t-test (R package pwr) with a power of 0.8 and an alpha of 0.05/3 (multiple testing correction), allowing for 15% loss of participants due to various reasons. All participants had normal or corrected-to-normal vision, no history of psychiatric or neurological diseases, and were naive to the specific purpose of the experiments. All participants gave written informed consent to procedures approved by the Institutional Review Board of Santa Lucia Foundation (protocol no. CE/progr454), in conformity with the Declaration of Helsinki regarding the use of human participants in research.

### Apparatus and visual stimuli

Participants sat on a chair with an adjustable backrest, so that the head and torso were tilted backwards at 13° relative to the vertical (measured with a plumb line), resulting in a comfortable posture for all of them. They wore a head-mounted display (HMD) (Oculus Rift DK2; Oculus VR, LLC, Menlo Park, CA, USA), where a virtual scene was rendered three-dimensionally (3D) on a PC by XVR software (eXtreme Virtual Reality; VRMedia s.r.l., Pisa, Italy). Visual stimuli were shown stereoscopically with the HMD. Each screen of the HMD had a resolution of 960 × 1080 pixels, a refresh rate of 75 Hz, and a diagonal field of view of 1.75 rad. Participants held an instrumented plastic pointer in their right hand. Four reflective passive spherical markers were attached to the HMD front to update the virtual scene based on head position and orientation, and four markers were attached on the hand pointer allowing its rendering in the virtual scene. In separate tests, we measured an average update latency of 3 stereo frames. The time-varying positions of the HMD and pointer markers in 3D space were acquired using a Vicon system (Oxford Metrics, Oxford, UK) equipped with 10 Bonita cameras (250 Hz sampling frequency). The 3D position of the midpoint between the eyes was derived from the position of the HMD markers.

The virtual scene depicted the laboratory (6.5 m wide, 18 m long, 5.0 m high in world scale) where the experiment took place, with desks and static human characters placed in different locations to provide cues about the reference and approximate metric scale of the scene (Fig. 1a,b). The scene was projected at a 1:1 scale, with truthful width-depth rendering. Perspective geometry, textures, directional lights, and shadows were included in the scene to augment 3D effects. Overhead lighting was provided. A green fixation cross was projected in the scene, and was located in the sagittal plane of the observer’s head, at a horizontal distance of 13.8 m and 3.2 m above the observer’s eyes. In the following, we define the fixation line as the sightline between the eyes and the fixation cross. Given the head orientation, the fixation cross was in the center of the screen along the sightline. In each trial, a textured ball (r = 0.045 m, referred to as “target” in the following) approached the observer frontally along parabolic paths, arriving in the same final position at a distance of 0.5 m from the observer’s eyes (Fig. 1a). Each spatial path (conditions #1—#7) was consistent with the effects of gravity (neglecting air drag), but only the trajectories whose kinematics obeyed gravitational acceleration were physically correct. The equations of motion are reported in the Supplementary Appendix. Each path was derived from Eq. A3 and one of 7 motion durations D, equally spaced (by 0.1 s) within the range 0.7–1.3 s (Table 1). For each parabola, the starting position and the final position of the target were aligned at the same height as the participant's eyes in all conditions (Fig. 1a). The maximum elevation angle of the target relative to the fixation line ranged between 0.29 and 0.37 rad across conditions. In particular, the maximum elevation angle was always smaller than the vertical field of view (± 0.58 rad), so that participants could see the entire target motion without moving the head. Because in each trial the stimulus duration co-varied with the initial horizontal distance from the observer (see Table 1), in theory participants could rely on either time or distance estimates to respond. Therefore, we also included two other conditions as a control for the effect of the initial target distance. The spatial paths of conditions #8 and #9 obeyed Eq. A1, and their distance was similar to that of two experimental conditions (condition #3 with duration 0.9 s and condition #5 with duration 1.1 s, respectively), but the control stimuli #8 and #9 had the same motion duration as the reference stimulus (1 s). The rationale for these control conditions was that, if participants used initial distance instead of duration, the responses for condition #8 (or #9) should be similar to those for condition #3 (or #5).

**Table 1:** Experimental (#1-#7) and control (#8, #9) conditions Condition #4 is the reference. v_x0_ and v_y0_ are the initial horizontal and vertical velocity, respectively.

Condition number	Horizontal distance [m]	Motion duration [s]	v_x0_ [m/s]			v_y0_ [m/s]		
			Const speed	Const vert	gravitational	Const speed	Const vert	gravitational
#1	1.31	0.700	1.28	0.94	1.87	2.34	1.72	3.43
#2	2.01	0.800	1.81	1.26	2.52	2.82	1.96	3.92
#3	2.82	0.900	2.32	1.57	3.13	3.26	2.21	4.41
#4	3.71	1.000	2.81	1.86	3.71	3.70	2.45	4.90
#5	4.7	1.100	3.27	2.14	4.27	4.12	2.70	5.39
#6	5.79	1.200	3.73	2.41	4.82	4.55	2.94	5.88
#7	6.96	1.300	4.16	2.68	5.36	4.94	3.19	6.37
#8	2.83	1.000	1.97	1.42	2.83	3.41	2.45	4.90
#9	4.55	1.000	3.64	2.28	4.55	3.92	2.45	4.90

**Figure 2:**
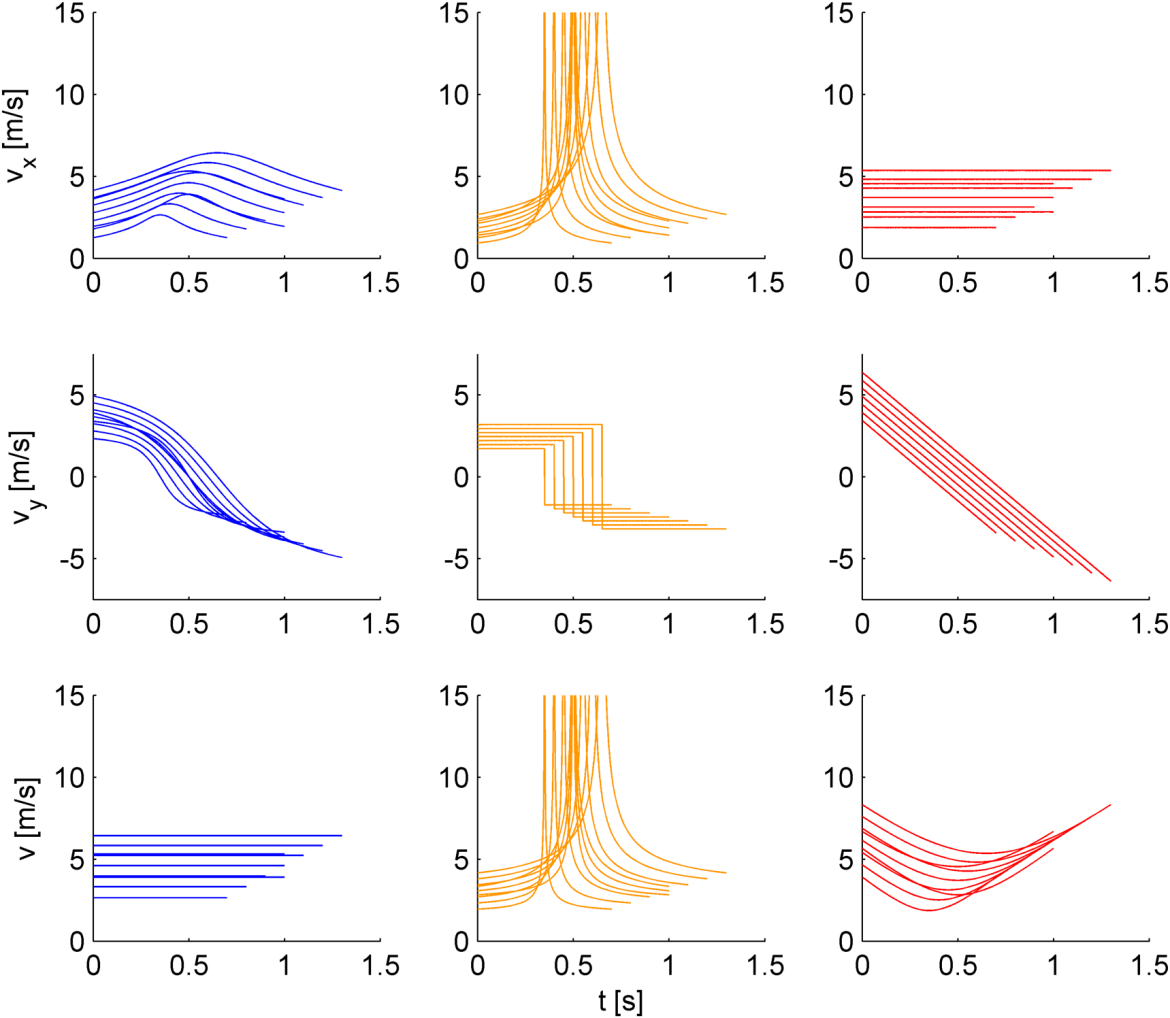
Time course of horizontal velocity (v_x_), vertical velocity (v_y_) and tangential velocity (v) for conditions #1-#9. Blue, orange and red curves correspond to the three laws of motion, constant tangential velocity, constant vertical velocity, and gravitational acceleration, respectively. The different lines in each panel correspond to the different conditions of motion duration.

**Figure 3:**
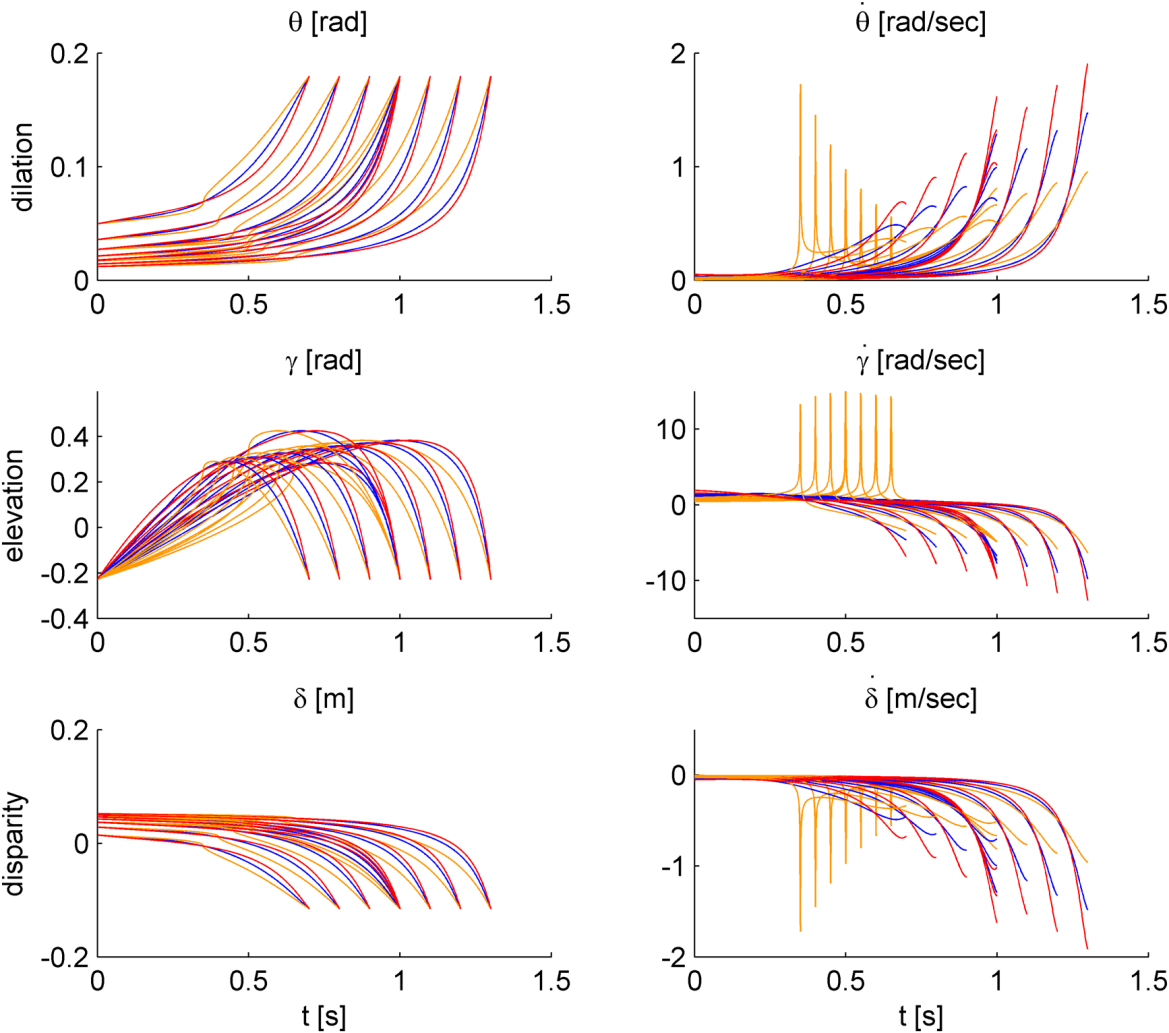
Time course of changes of dilation (retinal image expansion), dilation rate, elevation angle, elevation angle rate, disparity and changing disparity of the stimuli for conditions #1-#9. Blue, orange and red curves correspond to constant tangential velocity, constant vertical velocity, and gravitational acceleration, respectively.

In different trials, the target shifted on a given parabolic path with one of 3 different laws of motion: constant tangential velocity, constant vertical velocity (in absolute value), or gravitational acceleration (see Supplementary Appendix). The target motion duration on a given path was the same independently of the law of motion (see Table 1). Figure 2 shows the time course of the horizontal velocity (v_x_), vertical velocity (v_y_) and tangential velocity (v) for all stimuli. Figure 3 shows the time course of the changes of image dilation (θ), elevation angle (γ), binocular disparity (δ), as well as their time derivatives for all stimuli (assuming fixation of the cross). We defined a discriminability index (DI) for the rate of change of elevation angle as:

1 $${DI}_{\dot{\gamma }}=\frac{abs({\dot{\gamma }}_{comp}-{\dot{\gamma }}_{ref})}{{\dot{\gamma }}_{ref}}$$

where ${\dot{\gamma }}_{comp}$ and ${\dot{\gamma }}_{ref}$ are the rate of change of elevation angle for the comparison stimulus and the reference stimulus, respectively, and *abs* denotes the absolute value. This DI was computed at both the starting position of the target and when it intersected the fixation line for each parabola. Low values of DI would derive from small differences between comparison and reference stimuli as compared to the reference, indicating that the two stimuli may not be discriminable based on the rate of change of elevation angle^29^. The opposite would apply for high values of DI.

### Experimental Protocol

After the instructions, participants familiarized themselves with the virtual environment. All of them reported correct vision in the 3D environment by confirming that they saw the ball and the scene in 3D (instead of seeing two different images). Before each trial, the participants were reminded to keep fixation on the fixation cross throughout the trial. Although our HMD did not allow monitoring eye position, the participants reported that they were able to maintain fixation. Each trial consisted of a reference and a comparison stimulus (inter-stimulus interval = 500 ms), with the presentation order randomized across the trials. In each trial, after target disappearance, a written question appeared in the scene asking the participant to report whether the target moved for a longer time during the first or the second stimulus interval. To this end, two cubic white selection boxes (10 cm sides) were displayed in the virtual scene. The two boxes were placed at 15 cm and 35 cm, respectively, to the right of the midpoint position between the participant’s eyes. Both boxes were placed 40 cm below and 50 cm in front of the participant’s eyes. All faces of the left and right box were labeled with the number “1” and ”2”, respectively. By moving the hand with the pointer inside the white box with number “1” (or “2”), the participant indicated that she or he had perceived the first (or second) stimulus as lasting longer than the second (or first) one. Once selected, the box changed color (from white to gray) to signal the acquisition of the participant’s response, and the target appeared in the starting position of the next trial. The next trial started 500 ms after the previous response. The motion duration of the reference stimulus was 1 s, whereas the duration of the comparison stimuli varied between 0.7 and 1.3 s (Table 1). In each trial, the law of motion of the target (constant tangential velocity, constant vertical velocity, or gravitational acceleration) was the same for the reference and the comparison stimulus. No feedback about the correctness of responses was provided. Each combination of the laws of motion (n = 3) and parabolic paths (n = 9) was replicated 15 times in a pseudo-random order, resulting in 405 trials for each experiment. In the log file of each trial, we recorded the participant’s answer and the response time, which corresponded to the time interval between the arrival of the target of the second stimulus and the arrival of the pointer in the response box (“1″ or “2″). We allowed a maximum response time of 10 s.

### Statistical analysis

We excluded a few trials (3.4% of all trials) from the analysis when the response times were < 0 ms (indicating that the response occurred before the end of the second stimulus), > 10 s, or when the participant did not pay attention (as marked in the experiment notebook). Statistical analyses were performed in R^30^. The main statistical analyses involved the trials of conditions #1—#7 (see Table 1). The trials of conditions #8 and #9 were considered in a separate analysis, as a control for the effect of horizontal travelled distance. We fitted the results of each participant for each law of motion by using the psychometric function (General Linear Model):

2 $${\Phi }^{-1}[P\left(Y=1)\right]={\upeta }_{0}+{\upeta }_{1}\mathrm{D}$$

where P(Y = 1) is the probability of reporting that the comparison stimulus was longer than the reference stimulus, ${\Phi }^{-1}$[⋅] is the logit link function, and D is the motion duration of the comparison. The parameters η_0_ and η_1_ are the intercept and the slope of the general linear model, respectively. We estimated the point of subjective equivalence (PSE = −η_0_/η_1_) and the just noticeable difference (JND = log(3)/η_1_) from Eq. (2) (see^31^). We used the JND to evaluate the precision of the responses. The Weber Fraction is WF = JND/T_ref_, where T_ref_ is the duration (1 s) of the reference stimulus, so in our case JND = WF. Outlier participants were identified by applying the R function identify_outliers [rstatix package] to the JND of individual participants. Accordingly, when JND > (Q3 + 1.5 × IQR) or JND < (Q1 − 1.5 × IQR), the participant was defined as an outlier. Q1 and Q3 are the first and third quartiles, respectively. IQR is the interquartile range (IQR = Q3 − Q1). We used the Shapiro–Wilk test to verify the normality of distribution of JND or PSE data. Statistical differences between conditions were assessed using repeated-measures analysis of variance (RM-ANOVA) with the law of motion as within-subjects factor. Whenever RM-ANOVA detected a significant difference (alpha = 0.05), we performed multiple two-sided paired t-tests between the levels of the within-subjects factor. P-values were corrected for multiple comparisons by means of the Holm-Bonferroni method (using the R function pairwise_t_test).

Population responses were also analysed by means of a Generalized Linear Mixed Model (GLMM) that separately accounts for random effects due to inter-subject variability and fixed effects due to the experimental variables^31^. To this end, we fitted the data from the different participants and conditions with the following GLMM, selected from a pool of nested models based on the Bayesian information criterion (BIC):

3 $${\Phi }^{-1}\left[P\left(Y=1)\right]={\upbeta }_{0}+{\mathrm{u}}_{0}+{(\upbeta }_{1}+{\mathrm{u}}_{1}\right)\mathrm{D}+{(\upbeta }_{2}){\mathrm{M}}_{1}+{(\upbeta }_{3}){\mathrm{M}}_{2}+{(\upbeta }_{4}){\mathrm{DM}}_{1}+{(\upbeta }_{5}){\mathrm{DM}}_{2}$$

β_i_ are the fixed-effect parameters, while u_i_ are the random-effect parameters. D and M are the multiple predictor variables, D is the motion duration as a continuous variable and M_i_ the law of motion as dummy variable, DM_i_ are the corresponding interaction terms. We considered the condition of constant tangential velocity as the baseline condition, D was the motion duration of the comparison stimuli, M_1_ the constant vertical velocity condition and M_2_ the gravitational acceleration condition. Thus, β_0_ and β_1_ estimate the intercept and the slope for the baseline condition, (β_0_ + β_2_) and (β_1_ + β_4_) the intercept and the slope for the condition M_1_, and (β_0_ + β_3_) and (β_1_ + β_5_) the intercept and the slope for the condition M_2_. Notice that the results do not depend on the choice of the baseline condition.

We estimated the PSE (-intercept/slope) and the JND (log(3)/slope) from the GLMM (see^31^) for constant tangential velocity, constant vertical velocity, and gravitational acceleration. GLMM fitting was performed using the R packages lme4^32^ and MixedPsy^31^. We also employed a bootstrap method implemented in MixedPsy to test the differences between the different conditions. With this method, we computed the 95% confidence intervals of the difference of the JND (or PSE) between the experimental conditions (i.e., JND_ConstantTangentialVelocity_ − JND_ConstantVerticalVelocity_, JND_ConstantTangentialVelocity_ – JND_GravitationalAcceleration_, and JND_ConstantVerticalVelocity_ − JND_GravitationalAcceleration_). A 95% confidence interval that does not include zero implies that the tested conditions are significantly different.

As a control for the effect of horizontal distance (or initial position) of the target, we separately analyzed the trials of conditions #8 and #9 (see above) along with those of the experimental conditions with the same starting positions (#3 and #5). We selected the following GLMM from a pool of nested models based on BIC:

4 $${\Phi }^{-1}\left[P\left(Y=1)\right]={\upbeta }_{0}+{\mathrm{u}}_{0}+{(\upbeta }_{1}\right)\mathrm{H}+{(\upbeta }_{2})\mathrm{C}+{(\upbeta }_{3})\mathrm{HC}$$

As before, β_i_ are the fixed-effect parameters, while u_0_ is the random-effect parameter. H is the horizontal distance of the comparison stimulus (a continuous variable), and C is the dummy variable for the type of condition (0 for the experimental conditions and 1 for the control conditions). Here, β_0_ and β_1_ estimate the intercept and the slope for the baseline (the experimental conditions #3 and #5), and (β_0_ + β_2_) and (β_1_ + β_3_) estimate the intercept and the slope for the control conditions (#8 and #9). Thus, to evaluate whether participants used the initial horizontal distance to estimate motion duration, we compared the slope of the experimental conditions (${\upbeta }_{1})$ with that of the control conditions (${\upbeta }_{1}+{\upbeta }_{3})$. If participants used distance instead of duration to respond, we would expect ${\upbeta }_{3}=0$ (same slope for both conditions). Given that the motion duration of the control conditions was equal to the reference duration, if participants relied exclusively on duration estimates, we would expect ${\upbeta }_{3}={-\upbeta }_{1}$, i.e., slope = 0 for the conditions with the same duration independently of distance. Instead, if participants used both distance and duration, ${\upbeta }_{3}$ should be < 0.

## Results

In the experiments, the participants judged whether a ball approaching along a parabolic path took longer to arrive at destination in a 1-s reference trajectory or in a comparison trajectory whose duration randomly varied between 0.7 s and 1.3 s. Both the reference and the comparison obeyed one of three possible kinematic laws, randomly interleaved across trials: constant tangential velocity, constant vertical velocity (in absolute value), or gravitational acceleration.

**Figure 4:**
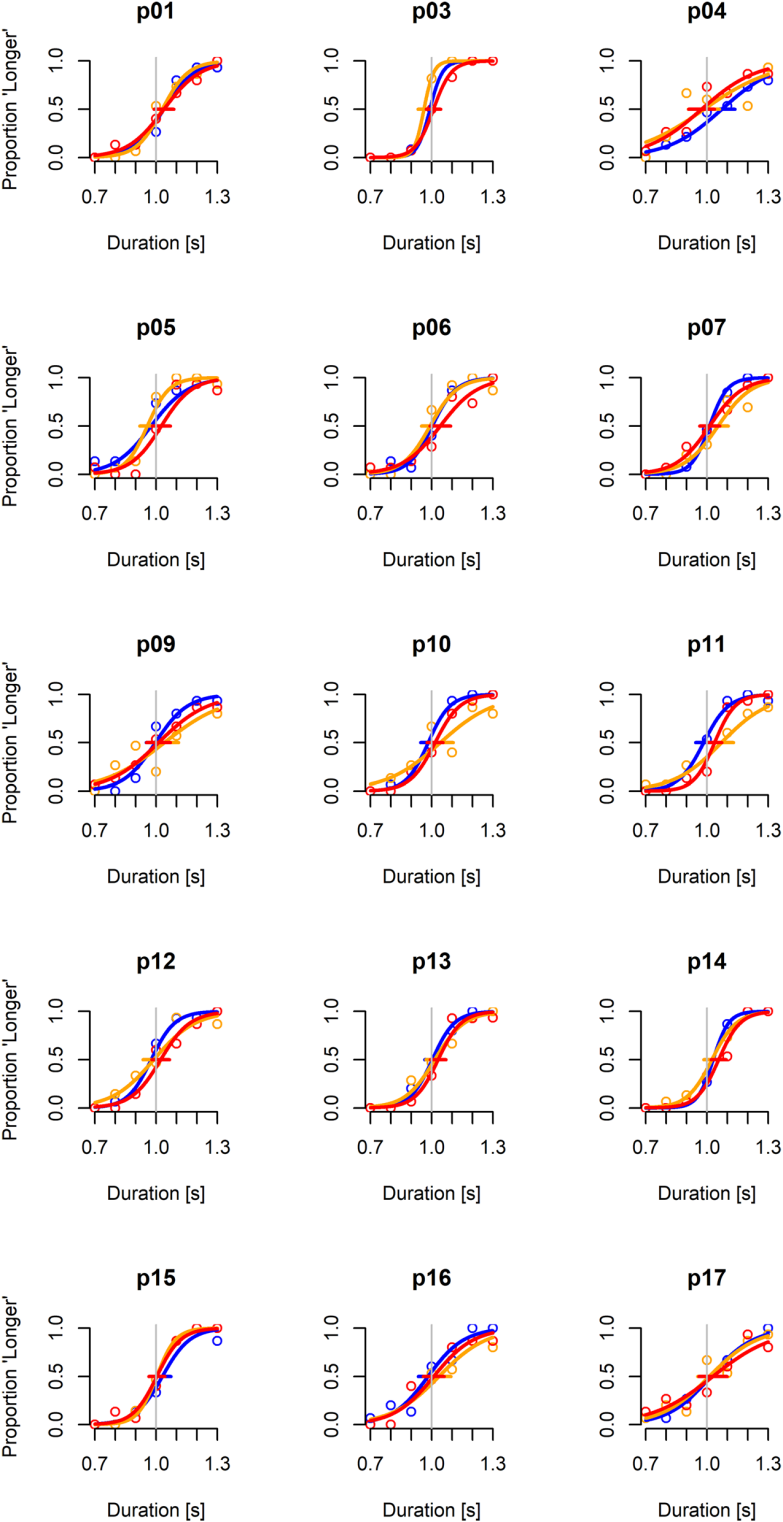
Psychometric functions for all participants (except the outliers) and laws of motion. Blue, orange and red curves correspond to constant tangential velocity, constant vertical velocity, and gravitational acceleration, respectively. Gray vertical line represents the reference duration (1 s).

**Figure 5:**
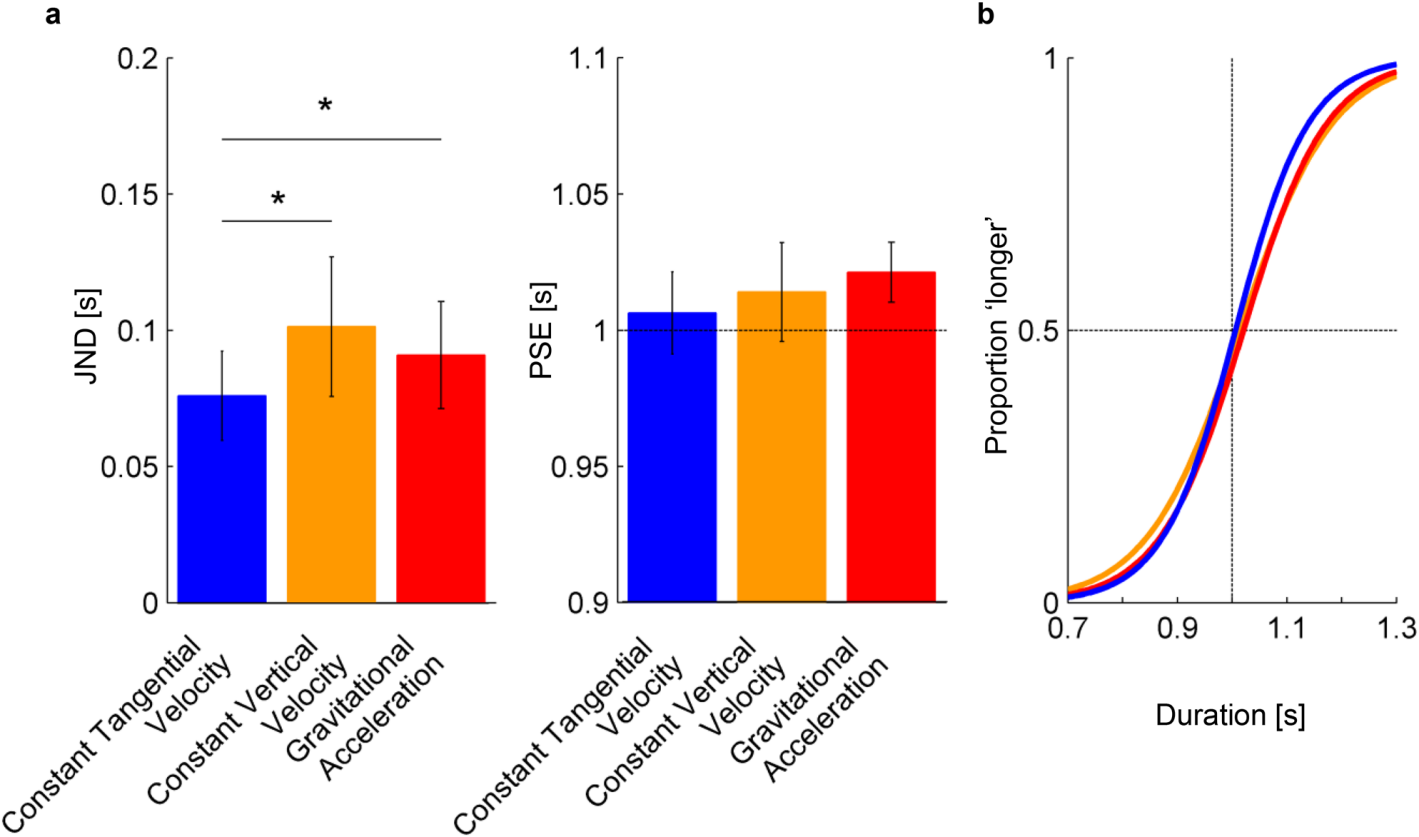
Panel a: JND and PSE for the three laws of motion (mean values and 95%-CI, n = 15 participants). For PSE, the dotted horizontal line denotes the duration of the reference stimulus (1 s). * P < 0.05 (corrected for multiple comparisons). Panel b: Psychometric functions predicted by the GLMM. Blue, orange and red curves correspond to constant tangential velocity, constant vertical velocity, and gravitational acceleration, respectively. The vertical line denotes the reference duration (1 s).

In the first pass of the analysis, we excluded participants p02 and p08 because their JND values were outliers (see Methods). The JNDs and PSEs of the other 15 participants were normally distributed for all 3 laws of motion of the target (all P-values > 0.145, Shapiro–Wilk test). The psychometric functions of each participant and each law of motion are plotted in Fig. 4. While these functions show some inter-subject variability, in all participants both the point of subjective equality (PSE) and the slope of the functions (whose inverse is the just noticeable difference, JND) varied little with the law of motion. However, in 12 out of 15 participants the slope was slightly higher for the constant tangential velocity than for the other 2 conditions, indicating a higher precision in the discrimination of the former condition. A RM-ANOVA over the responses of these 15 participants showed that the JND depended significantly on the law of motion (F(2,28) = 4.63, P = 0.018). The mean JND was equal to 0.076 s (95%-CI [0.059 s, 0.092 s]), 0.101 s (95%-CI [0.076 s, 0.127 s]), and 0.091 s (95%-CI [0.071 s, 0.111 s]), for the motion at constant tangential velocity, constant vertical velocity, and gravitational acceleration, respectively (Fig. 5a, left panel). These results indicate that the Weber Fractions were of the order of about 8–10% for the present conditions (see Methods). Post-hoc tests showed that the JND for the motion at constant tangential velocity was significantly smaller than that for the other two conditions (P < 0.05, after Holm-Bonferroni correction). The JND for the gravitational acceleration was not significantly different from that for the constant vertical velocity (P = 0.29).

The PSE did not depend significantly on the law of motion (F(2,28) = 1.1, P = 0.35). The mean PSE was equal to 1.006 s (95%-CI [0.991 s, 1.021 s]), 1.014 s (95%-CI [0.996 s, 1.032 s]), and 1.021 s (95%-CI [1.010 s, 1.032 s]) for the motion at constant tangential velocity, constant vertical velocity, and gravitational acceleration, respectively (Fig. 5a, right panel). Overall, this denotes that our motion manipulations did not bias the perceptual judgements. Moreover, the PSE was not significantly correlated with the JND: the linear regression between these two variables on the results pooled across participants and laws of motion had an R^2^ = 0.039 (adjusted for the number of predictor variables, P = 0.102), while the regression performed for each law of motion separately had an R^2^ < 0.105 (all P > 0.128).

When we included the two outlier participants (p02 and p08), the results were very similar to those described above (although noisier). The median JND over 17 participants was equal to 0.074 s (IQR [0.040 s]), 0.112 s (IQR [0.060 s]), and 0.089 (IQR [0.069 s]) for the motion at constant tangential velocity, constant vertical velocity, and gravitational acceleration, respectively. The median PSE was equal to 0.996 s (IQR [0.035 s]), 1.023 s (IQR [0.047 s]), and 1.022 s (IQR [0.026 s]) for the motion at constant tangential velocity, constant vertical velocity, and gravitational acceleration, respectively.

The GLMM analysis confirmed the previous results (Fig. 5b). In Eq. (3), the parameters β_4_ and β_5_ that account for the difference in slope between the baseline (constant tangential velocity) and the other two laws of motion (constant vertical velocity and gravitational acceleration) were statistically significant (P = 0.0002 and P = 0.028 respectively, Table 2) and negative, indicating a higher precision for the motion at constant tangential velocity. Using a bootstrap method (see Methods), we also found that both the difference between the JND for the constant tangential velocity and the JND for the constant vertical velocity, and the difference between the JND for the constant tangential velocity and the JND for the gravitational acceleration were significantly negative (-19.8 ms, 95%-CI [-31.7 ms, -9.4 ms]; -10.5 ms, 95%-CI [-20.9 ms, -2.3 ms], respectively). The difference between the JND for the constant vertical velocity and the JND for the gravitational acceleration was not significantly different from zero (-9.3 ms, CI [-20.3 ms, 3.0 ms]). The GLMM analysis also confirmed that the PSE did not depend on the law motion (the differences between all pairs of PSE were not significantly different from zero, as determined with bootstrapped 95% confidence intervals).

**Table 2:** All coefficients of the GLMM for the fixed factors in the model used to fit the probability of ‘longer’ responses for 15 participants and the relative P value are shown (Baseline: constant tangential velocity).*P < 0.05. **P < 0.01. ***P < 0.001.

		Estimate	Std. Error	z value	Pr( >|z|)
Baseline—constant tangential velocity:	Intercept (β_0_)	− 15.04	1.21	− 12.406	< 0.0001 ***
	Slope (β_1_)	14.94	1.20	12.459	< 0.0001 ***
Effect of constant vertical velocity on:	Intercept (β_2_)	3.10	0.82	3.761	< 0.0001 ***
	Slope (β_4_)	− 3.17	0.81	− 3.903	0.0002 ***
Effect of gravitational acceleration on:	Intercept (β_3_)	1.69	0.86	1.955	0.051
	Slope (β_5_)	− 1.87	0.85	− 2.203	0.028 *

Since in each trial the stimulus duration co-varied with the initial horizontal distance of the target from the observer, in theory the participants could rely exclusively on distance estimates to respond. To test for this possibility, we separately compared the results of two control conditions (#8 and #9, see Table 1) with those of two experimental conditions (condition #3 and #5) with a similar target distance but a different duration, since the control conditions had the same duration as the reference stimulus. The rationale for these control conditions was that, if participants used only distance to respond, the responses for condition #8 (or #9) should be similar to the responses for condition #3 (or #5). The results of the GLMM (Eq. (4), Table 3) ruled out this hypothesis, since the slope of the function for the experimental conditions was significantly higher than that for the control conditions: the slope was equal to 1.48 (95%-CI [1.24, 1.74]) and 0.67 (95%-CI [0.44, 0.92]) for the experimental and the control conditions, respectively. However, since the slope for the control conditions was significantly greater than zero, the results suggest that participants responded using distance to some extent, in addition to duration.

**Table 3:** All coefficients of the GLMM for the fixed factors in the model used to control the effect of the horizontal distance and the relative P value are shown (Baseline: experimental conditions).*P < 0.05. **P < 0.01. ***P < 0.001.

		Estimate	Std. Error	z value	Pr( >|z|)
Baseline—experimental conditions:	Intercept (β_0_)	− 5.72	0.50	− 11.447	< 0.0001 ***
	Slope (β_1_)	1.48	0.13	11.269	< 0.0001 ***
Effect of control experiment on:	Intercept (β_2_)	3.19	0.39	8.237	< 0.0001 ***
	Slope (β_3_)	− 0.81	0.10	− 8.096	< 0.0001 ***

Target speed is another parameter that observers might have used to respond, since it also co-varied with motion duration. In contrast with the initial target distance, initial target speed differed across laws of motion (see Table 1). We computed the putative JND of discrimination of initial tangential velocity by normalizing the velocity of each comparison stimulus with that of the corresponding reference stimulus. A RM-ANOVA over the responses of 15 participants showed that this JND did not depend significantly on the law of motion (F(2,28) = 1.58, P = 0.22). The same result was obtained using GLMM. We also performed a similar analysis considering the optic variables of the rate of change of either image dilation or elevation angle (Fig. 3). For this analysis, we considered the values of the optic variables at the start of motion, at the intersection with the fixation line during the ascending phase of the parabola, at the intersection with the fixation line during the descending phase, and at destination (see Fig. 1a). None of these putative JND depended significantly on the law of motion (RM-ANOVA, all P > 0.05), except for the JND of the rate of change of elevation angle at the start of motion (F(2,28) = 7.27, P = 0.003). In this case, the JND for the condition at constant tangential velocity was significantly lower than that for the other 2 conditions (P < 0.05, after Holm-Bonferroni correction).

The average response time relative to the arrival of the target of the second stimulus was 1.31 s (95%-CI [1.09 s, 1.53 s]), and did not depend significantly on any factors (law of motion, duration, repetition) or interaction (all P-values > 0.19).

## Discussion

We compared the discrimination of duration of different types of approaching parabolic motion, i.e., motion at constant tangential velocity, constant vertical velocity, or gravitational acceleration. Only the last motion was fully consistent with Earth gravity constraints, whereas the motions with constant velocity profiles obeyed the spatio-temporal constraint of parabolic paths dictated by gravity but violated the kinematic constraint. We found that, on average, the discrimination of duration was accurate and precise for all types of motions, but the discrimination of the trajectories at constant tangential velocity was slightly but significantly more precise than that of the trajectories at constant vertical velocity or gravitational acceleration. However, the implicit discrimination of tangential velocity and of the rate of change of optic variables (image dilation, elevation angle) did not depend significantly on the law of motion. Thus, we reject the hypothesis of a quantitative model of gravitational kinematics for the present task, the results being compatible with a heuristic internal model that is engaged by the view of projectiles shifting along gravitational parabolic paths irrespective of the specific kinematics.

### Discrimination precision

The Weber Fractions of duration discrimination ranged between about 8% and 10%. These values are comparable to or better than those reported in previous studies involving the discrimination of duration of targets falling vertically^23–25^, despite the fact that the targets moved in the fronto-parallel plane in the previous studies while they moved in stereoscopic view in the sagittal plane in the present study. This result is somewhat surprising since it is known that looming stereomotions are generally harder to perceive than the lateral motion equivalents (e.g.^33–36^). The present Weber Fractions are considerably better than those (between 15 and 30%, with high between-participant variability) reported for the discrimination of different gravity levels (between 0.7 g and 1.3 g) for virtual spheres approaching frontally on parabolic trajectories viewed stereoscopically^37^. They are also better than those (> 20%) reported for speed change discrimination of motion in depth^28^, or those (> 17%) reported for duration discrimination of static stimuli with the same duration (1 s) as the present reference stimuli^38,39^.

The good precision of duration discrimination that we found depended presumably on the opportunistic use of multiple cues^40,41^. In theory, our participants could respond by using estimates of the initial distance of the target, height of the parabola vertex, and/or target speed, in addition to the estimate of motion duration. It should be noted, however, that distance and speed can be systematically underestimated in virtual reality^42^. Also the optical variables could carry relevant information. Indeed, simulation studies by Gomez and López-Moliner^43^ showed that the time-to-contact of a parabolic target approaching the observer can be encoded by a combination of image dilation and elevation angle, provided the target size is known a priori (see also^37^). Specifically, the flight duration T_flight_ of a ball approaching the observer with parabolic motion can be specified by^43^:

5 $${\mathrm{T}}_{\mathrm{flight}}=\frac{\mathrm{s}}{{\mathrm{v}}_{\mathrm{r}}\uptheta +{\mathrm{tan}}(\upgamma ){\mathrm{s}}\dot{\gamma }}$$

where s is the ball size, θ the visual angle of the ball, γ the elevation angle, $\dot{\gamma }$ the rate of change of elevation angle, and v_r_ the radial velocity of the ball. Equation 5 includes optical variables (θ, γ, $\dot{\gamma }$) and physical variables (s, v_r_). Optical variables are available from optic flow cues during fixation. As for the physical variables, ball size was constant throughout the present experiments; although not known a priori, it could be roughly estimated by comparison with familiar objects and persons in the virtual scene (see Figs. 1a-b). It is also known that only few trials are needed to accurately estimate size when stereoscopic information is available and the simulated physical size is reliable^44^, as was the case in our experiments. Radial velocity v_r_ carries the isotropic expansion of the projected image on the retina, but it is not easily accessible from visual information. Its recovery would also depend on the knowledge of target size^43^, and might be facilitated under special conditions of target speed (see below).

To address the issue of the potential role of critical optic variables, we computed a discriminability index (DI) of the rate of change of elevation angle at both the starting position of the target and when it intersected the fixation line (see Methods). We found that, for all parabolas and laws of motion, the DI was always higher than 9% and reached values up to about 60%, indicating that the comparison stimulus and the reference stimulus were well discriminable based on the rate of change of elevation angle. Moreover, for large portions of the trajectories, the rate of change of elevation angle was within the theoretical optimal range of speed discrimination, the Weber Fraction of which has been shown to be about 7%^29^).

The availability of all these visual cues may explain the precision of discrimination for all laws of motion, but it does not explain why the duration of motions at constant tangential velocity was discriminated slightly but significantly better than those at constant vertical velocity or gravitational acceleration. Indeed, the general cues of initial horizontal distance of the target, vertex of the parabola, and average speed were exactly matched across conditions. Also, the changes in optical variables were roughly similar across conditions, except for the singularity at mid-trajectory for the constant vertical velocity (see Fig. 3). If anything, the variations of the time derivatives of image dilation, elevation angle and disparity over the whole path were larger and potentially more salient for the motions at gravitational acceleration. Accordingly, one would expect that the latter condition should have yielded better discriminations than the others, which was not the case.

One possibility for the constant tangential velocity condition showing higher precision is related to the computation of the flight duration based on Eq. (5). As remarked above, the use of this equation resorts to knowing v_r_, which is not easily accessible from visual information^43^. However, v_r_ is the radial component of the tangential velocity, and keeping tangential velocity constant would make the recovery of v_r_ easier (less noisy) for a given parabolic path.

Another related explanation for the superior duration discrimination of motions at constant tangential velocity is that these motions are computationally easier to track in the coordinates of Cartan’s moving frames attached to the target, which are the frames frequently used in computer vision^45^. In Cartan’s frames, magnitude and directional information of a motion are separated^46^. For the present parabolic trajectories, directional information was specified by the spatio-temporal constraint dictated by gravity. The amplitude (magnitude) of tangential velocity in world coordinates was constant throughout only in the case of constant tangential velocity.

A different possibility is that the trajectories with constant tangential velocity might be reminiscent of projectile motions affected by both gravity and air drag. It is known that a real, lightweight ball, with size roughly comparable to that of the present experiments and launched across distances comparable to our longest parabolas, experiences significant effects of air drag^47^. Our virtual reality scenarios provided only approximate cues about the size and texture of the ball, and no cues whatsoever about ball mass. Thus, the observers could not assess the effects of air drag, but we cannot exclude that they implicitly expected some drag effects for the simulated motions^7^. The presence of drag would distort the parabolic paths, but such deviations might not be perceived on the optical projection. On the other hand, the horizontal velocity smoothly decreased in the second half of the trajectories with constant tangential velocity (see Fig. 2), reproducing in part velocity changes resulting from drag.

## Conclusions

We showed that the durations of the parabolic motions with gravitational acceleration were not discriminated better than those with non-gravitational kinematics. This suggests that the subjective estimates of relative motion duration did not involve the quantitative value of gravitational acceleration. The present results indicate that, at least in the case of discrimination of motion duration, the gravity prior defines the spatio-temporal trajectory expected under the effects of Earth gravity, but does not specify the exact kinematics. This is reminiscent of the Intuitive Physics Engine proposed by Smith et al.^5^ to account for various kinds of interactions with projectile motion in the fronto-parallel plane, where participants extrapolate the path they believe the ball will take by taking into account the curvature due to gravity. Heuristics uses simple models that only approximate physical reality. This kind of internal model of gravity effects would provide heuristic templates to interpret visible trajectories, rather than quantitative predictions consistent with Newtonian models^48^. Any projectile motion along a parabolic (or quasi-parabolic) path temporally consistent with gravity would be interpreted as affected by gravity. Indeed, a quantitative representation of target kinematics would involve computation of many variables, and it is not at all clear that such a complex computation would increase performance by a useful amount. Rather, a general heuristic (a template) might provide information that is generally good enough, while requiring much less cognitive processing or visual resources. However, gravity effects may be anticipated even when both spatial and temporal constraints of physical gravity are violated by the visual stimuli. Thus, when projectiles are thrown at constant speed along a sloping linear path instead of a gravitational parabola, subjects tend to place their hand closer to the expected parabolic path than the visible linear path^11^. At the cognitive level, the final position of a horizontally moving target that is suddenly halted is misremembered as being displaced downward below the path of motion, consistent with the idea that gravity effects are implicitly assumed by the observers (so-called representational gravity^17^.

## Supplementary Information

Supplementary Information

## Data Availability

Source data are deposited at https://zenodo.org/record/4271547.

## References

[1] Shepard RN (1994). Perceptual-cognitive universals as reflections of the world. Psychon. Bull. Rev..

[2] Shepard, R. N. (1981). “Psychophysical complementarity,” in Perceptual Organization, eds. M. Kubovy and J. R. Pomerantz (Hillsdale, NJ: Erlbaum), pp. 279–341

[3] Hegarty M (2004). Mechanical reasoning by mental simulation. Trends Cogn Sci.

[4] Kaiser MK, Proffitt DR, McCloskey M (1985). The development of beliefs about falling objects. Atten. Percept. Psychophys..

[5] Smith KA, Battaglia PW, Vul E (2018). Different physical intuitions exist between tasks, not domains. Comput. Brain Behav..

[6] Zago M, Lacquaniti F (2005). Cognitive, perceptual and action-oriented representations of falling objects. Neuropsychologia.

[7] D'Andola M, Cesqui B, Portone A, Fernandez L, Lacquaniti F, d'Avella A (2013). Spatiotemporal characteristics of muscle patterns for ball catching. Front. Comput. Neurosci..

[8] Delle Monache S, Lacquaniti F, Bosco G (2019). Ocular tracking of occluded ballistic trajectories: Effects of visual context and of target law of motion. *J. Vis.* 19(4):13, 1–21. 10.1167/19.4.13 30952164

[9] Fiehler K, Brenner E, Spering M (2019). Prediction in goal-directed action. J. Vis..

[10] Jörges B, López-Moliner J (2019). Earth-gravity congruent motion facilitates ocular control for pursuit of parabolic trajectories. Sci Rep.

[11] Russo M, Cesqui B, La Scaleia B, Ceccarelli F, Maselli A, Moscatelli A, Zago M, Lacquaniti F, d'Avella A (2017). Intercepting virtual balls approaching under different gravity conditions: evidence for spatial prediction. J. Neurophysiol..

[12] Fink PW, Foo PS, Warren WH (2009). Catching fly balls in virtual reality: A critical test of the outfielder problem. J. Vis..

[13] McLeod P, Reed N, Dienes Z (2006). The generalized optic acceleration cancellation theory of catching. J. Exp. Psychol. Hum. Percept. Perform..

[14] Zago M, McIntyre J, Senot P, Lacquaniti F (2009). Visuo-motor coordination and internal models for object interception. Exp. Brain Res..

[15] La Scaleia B, Zago M, Lacquaniti F (2015). Hand interception of occluded motion in humans: a test of model-based vs. on-line control. J. Neurophysiol..

[16] López-Moliner J, Brenner E, Louw S, Smeets JB (2010). Catching a gently thrown ball. Exp. Brain Res..

[17] Hubbard TL (2020). Representational gravity: Empirical findings and theoretical implications. Psychon. Bull. Rev..

[18] Brouwer AM, López-Moliner J, Brenner E, Smeets JB (2006). Determining whether a ball will land behind or in front of you: Not just a combination of expansion and angular velocity. Vis. Res..

[19] Hecht H, Bertamini M (2000). Understanding projectile acceleration. J. Exp. Psychol. Hum. Percept. Perform..

[20] Reed N, McLeod P, Dienes Z (2010). Implicit knowledge and motor skill: What people who know how to catch don’t know. Conscious. Cogn..

[21] Shaffer DM, McBeath MK (2005). Naive Beliefs in Baseball: Systematic Distortion in Perceived Time of Apex for Fly Balls. J Exp Psychol Learn Mem Cogn.

[22] Todd J (1981). Visual information about moving objects. J Exp Psychol Hum Percept Perform.

[23] Gallagher M, Torok A, Klaas J, Ferrè ER (2020). Gravity prior in human behaviour: a perceptual or semantic phenomenon?. Exp Brain Res.

[24] Moscatelli A, Lacquaniti F (2011). The weight of time: gravitational force enhances discrimination of visual motion duration. J. Vis..

[25] Torok A, Gallagher M, Lasbareilles C, Ferrè ER (2019). Getting ready for Mars: How the brain perceives new simulated gravitational environments. Q. J. Exp. Psychol. (Hove).

[26] Cesqui B, Mezzetti M, Lacquaniti F, d'Avella A (2015). Gaze Behavior in One-Handed Catching and Its Relation with Interceptive Performance: What the Eyes Can't Tell. PLoS ONE.

[27] Cormack LK, Czuba TB, Knoll J, Huk AC (2017). Binocular Mechanisms of 3D Motion Processing. Annu. Rev. Vis. Sci..

[28] Lee ARI, Ales JM, Harris JM (2019). Speed change discrimination for motion in depth using constant world and retinal speeds. PLoS ONE.

[29] De Bruyn B, Orban GA (1988). Human velocity and direction discrimination measured with random dot patterns. Vis. Res..

[30] R Core Team (2018). R: A language and environment for statistical computing.

[31] Moscatelli A, Mezzetti M, Lacquaniti F (2012). Modeling psychophysical data at the population-level: the generalized linear mixed model. J. Vis..

[32] Bates D, Machler M, Bolker B, Walker S (2015). Fitting Linear Mixed-Effects Models Using lme4. J. Stat. Softw..

[33] López-Moliner J, Maiche A, Estaún i Ferrer, S, (2003). Perception of acceleration in motion-in-depth with only monocular and both monocular and binocular information. Psicológica.

[34] Regan D, Beverley KI (1979). Binocular and monocular stimuli for motion in depth: Changing-disparity and changing-size feed the same motion-in-depth stage. Vis. Res..

[35] Tyler CW (1971). Stereoscopic depth movement: two eyes less sensitive than one. Science.

[36] Westheimer G (1990). Detection of disparity motion by the human observer. Optom. Vis. Sci..

[37] Jörges B, Hagenfeld L, López-Moliner J (2018). The use of visual cues in gravity judgements on parabolic motion. Vis. Res..

[38] Jones LA, Poliakoff E, Wells J (2009). Good vibrations: Human interval timing in the vibrotactile modality. Q. J. Exp. Psychol..

[39] Rammsayer T, Pichelmann S (2018). Visual-auditory differences in duration discrimination depend on modality-specific, sensory-automatic temporal processing: Converging evidence for the validity of the Sensory-Automatic Timing Hypothesis. Q. J. Exp. Psychol..

[40] Eagleman DM (2008). Human time perception and its illusions. Curr Opin Neurobiol.

[41] Lee ARI, Ales JM, Harris JM (2020). Three-Dimensional Motion Perception: Comparing Speed and Speed Change Discrimination for Looming Stimuli. Vision (Basel).

[42] Rolin RA, Fooken J, Spering M, Pai DK (2018). Perception of looming motion in virtual reality egocentric interception tasks. IEEE Trans. Visual Comput. Graphics.

[43] Gómez J, López-Moliner J (2013). Synergies between optical and physical variables in intercepting parabolic targets. Front Behav Neurosci.

[44] López-Moliner J, Keil M (2012). People favour imperfect catching by assuming a stable world. PLoS ONE.

[45] Paragios N, Chen Y, Faugeras OD (2006). Handbook of mathematical models in computer vision.

[46] Olver PJ (2005) A survey of moving frames. In: Li H, Olver PJ, Sommer G, eds. Computer Algebra and Geometric Algebra with Applications, Lecture Notes in Computer Science. New York: Springer-Verlag, Volume 3519. pp 105–138.

[47] D'Avella A, Cesqui B, Portone A, Lacquaniti F (2011). A new ball launching system with controlled flight parameters for catching experiments. J Neurosci Methods.

[48] Zago M, McIntyre J, Senot P, Lacquaniti F (2008). Internal models and prediction of visual gravitational motion. Vision Res.

