# Supplementary material for: Perceptual judgments of duration of parabolic motions
Source: Sci Rep. 2021 Mar 29;11:7108. doi: 10.1038/s41598-021-86428-3 (PMC8007634; doi:10.1038/s41598-021-86428-3)
Supplement: Supplementary file 1 — Supplementary Information [file 41598_2021_86428_MOESM1_ESM.docx]

**APPENDIX**

The equation of gravitational motion is

$\left\{ \begin{matrix} x\left( t \right)=v_{0x}t+x_{0} \\ y\left( t \right)=-\frac{1}{2} g t^{2}+v_{0y}t+y_{0} \end{matrix} \right.$ $0\leq t\leq D$ (A1)

where *x* and *y* are the horizontal and the vertical position, *x_0_*, *y_0_* the initial position, *v_0x_* and *v_0y_* the initial velocity, *g* the gravitational acceleration (9.81 m/s^2^), *t* the time, and D the overall motion duration for a given condition.

For each D, we imposed the following boundary conditions:

$\left\{ \begin{matrix} y\left( t=D \right)=y\left( t=0 \right)=y_{0} \\ x\left( t=D \right)=x_{end} \end{matrix} \right.$ (A2)

where (*x_end_*, *y_0_*) are the coordinates of the final position of the target (at a distance of 0.5 m from the observer’s eyes) and (*x _end_* – *x_0_*) the horizontal travelled distance.

By eliminating *t* between the two equations of A1, we obtain the spatial parabolic path:

$y\left( x \right)=-\frac{1}{2} g {\frac{(x-x_{0})}{{v_{0x}}^{2}}}^{2}+v_{0y}\frac{(x-x_{0})}{v_{0x}}+y_{0}$ (A3)

The target shifted along this path with one of 3 different laws of motion: gravitational acceleration (Eq. A1), constant tangential velocity, or constant vertical velocity.

In order to calculate the equation of motion for the condition with constant tangential velocity, we evaluated the length of the parabolic path:

$L\left( y,\left[ x_{0},x_{end} \right] \right)=\int_{x_{0}}^{x_{end}} \sqrt{1+{(\frac{dy}{dx})}^{2}}dx=\int_{x_{0}}^{x_{end}} \sqrt{1+{(ax+b)}^{2}}dx=\left. \frac{\sqrt{1+\left( ax+b \right)^{2}}*\left( ax+b \right)+\sinh^{-1}( ax+b)}{2a} \right]_{x_{0}}^{x_{end}}$ (A4)

where

$a=-\frac{g}{{v_{0x}}^{2}}; b=\frac{g x_{0}}{{v_{0x}}^{2}}+\frac{v_{0y}}{v_{0x}}$ (A5)

Next, we calculated the value of the constant tangential velocity as:

$v_{tan}=\frac{L}{D}$ (A6)

At time $t_{i}$ , the length of the travelled parabolic path at constant tangential velocity$v_{tan}$ is:

$l\left( t_{i} \right)=\frac{\sqrt{1+\left( ax_{i}+b \right)^{2}}*(ax_{i}+b)+\sinh^{-1}( ax_{i}+b)}{2a}-\frac{\sqrt{1+\left( ax_{0}+b \right)^{2}}*\left( ax_{0}+b \right)+\sinh^{-1} \left( ax_{0}+b \right)}{2a}=v_{tan}*t_{i}$ (A7)

Defining $c\left( x_{i} \right)= ax_{i}+b$ and $f=\frac{\sqrt{1+\left( c\left( x_{0} \right) \right)^{2}}* c\left( x_{0} \right)+\sinh^{-1} \left( c\left( x_{0} \right) \right)}{2a}$, equation A7 becomes:

$\sqrt{1+\left( c\left( x_{i} \right) \right)^{2}}*c\left( x_{i} \right)+\sinh^{-1}( c(x_{i}))=2a*( f+v_{tan}*t_{i})$ (A8)

With the numerical solution ($c\left( x_{i} \right)$) for each *t_i_*, we obtained the equation of motion for the condition with constant tangential velocity:

$\left\{ \begin{matrix} x\left( t_{i} \right)=\frac{c\left( x_{i} \right)-b}{a} \\ y\left( t_{i} \right)=-\frac{1}{2} g {\frac{\left( x\left( t_{i} \right)-x_{0} \right)}{{v_{0x}}^{2}}}^{2}+v_{0y}\frac{\left( x\left( t_{i} \right)-x_{0} \right)}{v_{0x}}+y_{0} \end{matrix} \right.$ $0\leq t_{i}\leq D$ (A9)

The equation of motion at constant vertical velocity was obtained by calculating the absolute value of vertical velocity as:

$v_{vert}=\Delta y*2*\frac{1}{D}=\frac{t_{1}^{2}*g}{2}*2*\frac{1}{D}$ (A10)

where $\Delta y=y_{max}-y_{0}$ is the difference between the greatest height (*y_max_*) that the target reaches (vertex of the parabola) and the initial height, and the *t_1_* = D/2 is the time to reach the maximum height.

$\left\{ \begin{matrix} y\left( t_{i} \right)=v_{vert}*t_{i}+y_{0} \\ x\left( t_{i} \right)=v_{0x} \frac{v_{oy}-\sqrt{{v_{0y}}^{2}-2g\left( y\left( t_{i} \right)-y_{0} \right)}}{g}+x_{0} \end{matrix} \right.$ $0\leq t\leq\frac{D}{2}$

(A11)

$\left\{ \begin{matrix} y\left( t_{i} \right)={y_{max}-v}_{vert}*(t_{i}-\frac{D}{2} ) \\ x\left( t_{i} \right)=v_{0x}\frac{v_{oy}+\sqrt{{v_{0y}}^{2}-2g(y\left( t_{i} \right)-y_{0})}}{g}+x_{0} \end{matrix} \right.$ $\frac{D}{2}<t\leq D$
